# Risk Stratification of Patients with Peripheral Arterial Disease and Abdominal Aortic Aneurysm Using Aortic Augmentation Index

**DOI:** 10.1371/journal.pone.0139887

**Published:** 2015-10-09

**Authors:** Marianne Beckmann, Vincenzo Jacomella, Malcom Kohler, Mario Lachat, Amr Salem, Beatrice Amann-Vesti, Marc Husmann

**Affiliations:** 1 Clinic for Angiology, University Hospital Zurich and University of Zurich, Zurich, Switzerland; 2 Clinic for Pneumology, University Hospital Zurich and University of Zurich, Zurich, Switzerland; 3 Clinic for Cardiovascular Surgery, University Hospital Zurich and University of Zurich, Zurich, Switzerland; 4 Medical Research Institute, Alexandria University, Alexandria, Egypt; 5 Angiology Division, Department of Internal Medicine, Kantonsspital St. Gallen, St Gallen, Switzerland; The University of Tokyo, JAPAN

## Abstract

**Background:**

Central augmentation index (cAIx) is an indicator for vascular stiffness. Obstructive and aneurysmatic vascular disease can affect pulse wave propagation and reflection, causing changes in central aortic pressures.

**Aim:**

To assess and compare cAIx in patients with peripheral arterial disease (PAD) and / or abdominal aortic aneurysm (AAA).

**Methods:**

cAIx was assessed by radial applanation tonometry (Sphygmocor) in a total of 184 patients at a tertiary referral centre. Patients were grouped as having PAD only, AAA only, or both AAA and PAD. Differences in cAIx measurements between the three patient groups were tested by non-parametric tests and stepwise multivariate linear regression analysis to investigate associations with obstructive or aneurysmatic patterns of vascular disease.

**Results:**

In the study sample of 184 patients, 130 had PAD only, 20 had AAA only, and 34 patients had both AAA and PAD. Mean cAIx (%) was 30.5 ± 8.2 across all patients. It was significantly higher in females (35.2 ± 6.1, n = 55) than males (28.4 ± 8.2, n = 129), and significantly higher in patients over 80 years of age (34.4 ± 6.9, n = 22) than in those under 80 years (30.0 ± 8.2, n = 162). Intergroup comparison revealed a significant difference in cAIx between the three patient groups (AAA: 27.3 ± 9.5; PAD: 31.4 ± 7.8; AAA & PAD: 28.8 ± 8.5). cAIx was significantly lower in patients with AAA, higher in patients with both AAA and PAD, and highest in patients with PAD only (*beta* = 0.21, *p* = 0.006).

**Conclusion:**

Non-invasive assessment of arterial stiffness in high-risk patients indicates that cAIx differs according to the pattern of vascular disease. Measurements revealed significantly higher cAIx values for patients with obstructive peripheral arterial disease than for patients with aneurysmatic disease.

## Introduction

Atherosclerotic disease is the leading cause of mortality and morbidity in the Western world. The term encompasses coronary artery disease, cerebrovascular disease, peripheral arterial disease and aortic aneurysm [1]. Peripheral arterial disease (PAD) leads to a 2–5 fold increase in cardiovascular mortality [2–3]. Likewise, patients suffering from an aneurysm of the abdominal aorta (AAA) have an elevated risk of cardiovascular morbidity and mortality, due to the risk factors and comorbidities associated with aneurysm, and the risk of aneurysm rupture which has a very high mortality rate [4–7]. In this study, we use the term PAD to refer to patients with stenotic or occlusive arterial disease of the lower extremities only. PAD is often associated with AAA [8].

The treatment of cardiovascular risk factors, such as arterial hypertension, is important in reducing morbidity and mortality in both PAD and AAA patients. Current guidelines for anti-hypertensive treatment are based on peripheral blood pressure measurements. However, in recent years central hemodynamic parameters have been ascribed an increasingly important role in the evaluation of cardiovascular risk. In particular, both increased central blood pressure and increased arterial stiffness have been shown to be independently associated with a poor cardiovascular outcome [9–12].

Arterial stiffness is a feature of both obstructive and aneurysmatic vascular diseases [13–14], and results from a loss of elasticity in the arterial wall. Currently, carotid-femoral pulse wave velocity (PWV) measurement is regarded as the gold standard method for assessment of arterial stiffness [15–16]. Other, indirect, measures of aortic stiffness include the central augmentation index (cAIx) [15]. In addition to pulse wave velocity, cAIx provides information on wave reflection patterns.

The pulse wave in any vascular segment is composed of a forward and backward wave, and its shape is dependent on the timing and magnitude of those two waves. The impact of the reflected wave is related to its early superimposition onto the forward wave and the magnitude and distribution of the reflected waves. Pressure waveforms can be recorded non-invasively by applanation tonometry. Different parameters have been defined in pulse wave analysis [16–18]. The central aortic augmentation index (cAIx) is defined as the pressure difference between the first and second systolic peaks (P2-P1 = Augmentation Pressure) expressed as a percentage of the pulse pressure (PP), which is the difference between diastolic and systolic blood pressure. The parameter provides an indication of the influence of reflected waves on the total pulse pressure. Apart from a high pulse wave velocity, changes in reflection sites can also influence the augmentation index. Both obstructive and aneurysmatic vascular disease patterns affect pulse wave propagation, due to changes in arterial wall characteristics.

To date, there has been no analysis directly comparing arterial stiffness parameters in these two disease patterns. We aimed to explore differences in arterial stiffness between obstructive and aneurysmatic disease patterns (patients with PAD, AAA or both) through non-invasive assessment of central hemodynamic markers. We used the cAIx recorded at the radial artery as our primary measure for arterial stiffness rather than carotid-femoral PWV, because aortic aneurysm and/or obstruction of the iliac arteries located in the direct axis for measurement of PWV would have influenced results.

## Methods

### Patient selection and study design

This was an open, non-randomized, comparative study conducted at a tertiary referral centre. The local ethics committee (Kantonale Ethikkommission Zürich, Stampfenbachstrasse 121, 8090 Zürich, Switzerland) approved the study (Nr. 1741/2009) and all patients gave written informed consent. The study was conducted according to Good Clinical Practice standards.

Data was collected and analysed from a total of 184 patients. Patients were divided into three groups: a) PAD only, b) AAA only, and c) patients with both AAA and PAD.

For all patients the following data was collected: medical history, brachial systolic and diastolic blood pressures, body mass index, vascular risk factors, comorbidities, medication, and radial artery pulse wave analysis as described below.

Patients were defined as having PAD if the ankle-brachial index (ABI) measurement was <0.9, or if ABI was >0.9 concomitant with a history of lower limb revascularization; patients had chronic and stable PAD that had been graded into Rutherford I-III according to their medical history. Measurements of cAIx in PAD patients were taken before any planned vascular intervention. Patients were defined as having AAA if an aneurysm of the abdominal aorta was present with a diameter of 3cm or greater, as described below.

All patients with AAA were evaluated for PAD based on information in their medical history and ankle-brachial pressure measurements. All PAD patients were screened for concomitant AAA using information acquired from their medical records, including existing ultrasound, CT and MRI images. The difference between the date of our cAIx measurements and the date of ultrasound, CT and MRI measurements was 24 months on average.

To verify our measurements for pulse wave analysis we performed measurements in a group of 18 healthy unmatched controls with no known cardiovascular risk factors (mean age 35 ± 10.7 years; female 66%, male 33%). For characteristics of this unmatched control group see S1 Table.

### Ankle-brachial arterial pressure index assessment

Ankle-brachial arterial pressure index (ABI) assessments were performed as part of the standard diagnostic procedure. Standard brachial systolic and diastolic blood pressures on both arms were measured in triplicate using a traditional cuff manometer, according to Riva Rocci methods. Systolic ankle blood pressures, of the posterior tibial artery and anterior tibial artery, on both legs, were obtained by hand-held 6 MHz Doppler probe (Kranzbühler, Logidop 2, Pilger Medical Electronics, Switzerland). For each leg, ABI was calculated as the ratio of the highest ankle systolic blood pressure to the highest brachial systolic blood pressure; the lower of these two ABI values was taken as the study parameter.

### Abdominal aortic aneurysm diameter measurements

The maximum abdominal aortic diameter was measured using ultrasound, CT or MRI angiography imaging techniques. The diameter of the abdominal aorta was defined as the maximum cross-sectional diameter (including the vessel wall), measured orthogonally to the estimated vessel centre line. Abdominal aortic aneurysm was defined as a diameter of 3cm or greater in the abdominal section of the infra-diaphragmatic aorta.

### Pulse wave analysis

Pulse wave analysis was conducted with applanation tonometry. All measurements were performed with the patient in the supine position. To record the central pressure waveform the indirect method of arterial tonometry was used: pressure waveform was recorded at the radial artery, and using the generalized transfer function it was converted into a calculated central pressure waveform [19]. All measurements were performed with the SphygmoCor device and designated software (AtCor Medical Pty. Ltd., Sydney, Australia). SphygmoCor uses a high fidelity Millar strain-gauge transducer (Millar Instruments, Houston, TX) allowing for measurement of the first systolic peak (P1), the second systolic peak (P2), and the central pulse pressure (PP) from the calculated aortic waveform. AIx was then calculated as:

AIx(%)=(P2−P1)PP*100 (1)

Because the heart rate is known to significantly affect AIx values, normalization was performed to a standard heart rate of 75bpm (Aix@75) [20]:

AIx@75=AIx−0.39*(75−HR)

### Statistical analysis

Statistical analyses were carried out using Stata/SE11.2 for Windows. Patient characteristics were presented as mean ± standard deviation or as absolute frequency and percent. The non-parametric Kruskal-Wallis test was used for comparison of mean cAIx between patient groups: first, differences in mean cAIx were analysed for age, gender, BMI and height; second, differences in mean cAIx were analysed by diagnosis (AAA only, PAD and AAA, and PAD only), also taking age and gender into account.

Multivariate regression analysis was applied, directly controlling for heterogeneities between patients. The dependent variable was cAIx@75. In a first model, cAIx in our patient groups was compared to cAIx in a control group of healthy individuals. Subsequently, cAIx was compared between patients grouped by diagnosis: the main explanatory variable, diagnosis, was defined as an ordinal variable taking the value 1 if a patient suffered from AAA, the value 2 if a patient suffered from AAA and PAD, and the value 3 if a patient suffered from PAD. In addition to the diagnosis the following control variables were included in four stepwise regressions: (i) age, gender, weight, height; then (i) and (ii) hypertension, smoking and diabetes; then (i) and (iii) coronary heart disease and cerebrovascular disease; and finally (i) and (iv) medication. It was not possible to control for medication and risk factors in the same specification as this introduced multi-collinearity. In order to check the robustness of our results, multivariate regression was repeated–first, by only including significant variables from the above four stepwise regressions, and second, by defining diagnosis as three binary variables.

## Results

### Patient characteristics

The study sample consisted of 184 patients: 71% (n = 130) had PAD only, 18% (n = 34) suffered from PAD and AAA and 11% (n = 20) had aortic aneurysm only.

Table 1 shows the patient characteristics for the three study groups. There was considerable heterogeneity within the respective groups of patients, particularly with regard to age and gender. In addition, the patients were shown to have cardiovascular comorbidities and cardiovascular risk factors known to be typical of patients with PAD and aneurysm.

**Table 1:** Patient Characteristics.

	AAA	PAD	AAA and PAD
	(n = 20)	(n = 130)	(n = 34)
Age, years	64.25 ± 8.08	68.70 ± 10.74	68.21 ± 5.77
Male, n (%)	18 (90)	82 (63)	29 (85)
Weight, kg	83.10 ± 11.65	72.63 ± 13.86	76.58 ± 11.57
Height, m	1.74 ± 0.09	1.68 ± 0.08	1.72 ± 0.08
BMI, kg/m^2^	27.60 ± 3.57	25.53 ± 4.08	25.89 ± 3.37
Waist, cm	101.75 ± 10.35	97.37 ± 12.52	101.75 ± 12.51
Hip, cm	104.90 ± 7.85	102.00 ± 8.11	103.69 ± 12.10
Waist-hip ratio	0.97 ± 0.07	0.95 ± 0.08	0.98 ± 0.08
**Cardiovascular Comorbidities**			
Coronary artery disease, n (%)	12 (60)	35 (27)	17 (50)
Cerebrovascular disease, n (%)	0 (0)	35 (27)	8 (24)
Renal insufficiency, n (%)	n/a	26 (20)	1 (9)
**Cardiovascular risk factors**			
Dyslipidemia, n (%)	14 (70)	81 (62)	30 (88)
Diabetes, n (%)	3 (15)	36 (28)	5 (15)
Current Smoking, n (%)	6 (30)	98 (75)	20 (59)
Arterial hypertension, n (%)	15 (75)	106 (82)	32 (94)
**Medication**			
Lipid lowering, n (%)	14 (70)	110 (85)	30 (88)
ACE inhibitor, n (%)	7 (35)	48 (37)	15 (44)
AT-II receptor blocker, n (%)	6 (30)	33 (25)	11 (32)
Aldosterone receptor antagonist, n (%)	0	5 (4)	3 (9)
β-blocker, n (%)	13 (65)	53 (41)	26 (76)
α1-adreno-receptor blocker, n (%)	2 (10)	1 (1)	1 (3)
Calcium channel blocker, n (%)	6 (30)	39 (30)	8(24)
Diuretics, n (%)	8 (40)	54 (42)	17 (50)
Nitrates, n (%)	1 (5)	4 (3)	1 (3)

Notes: n denotes the number of observations

**Fig 1:**
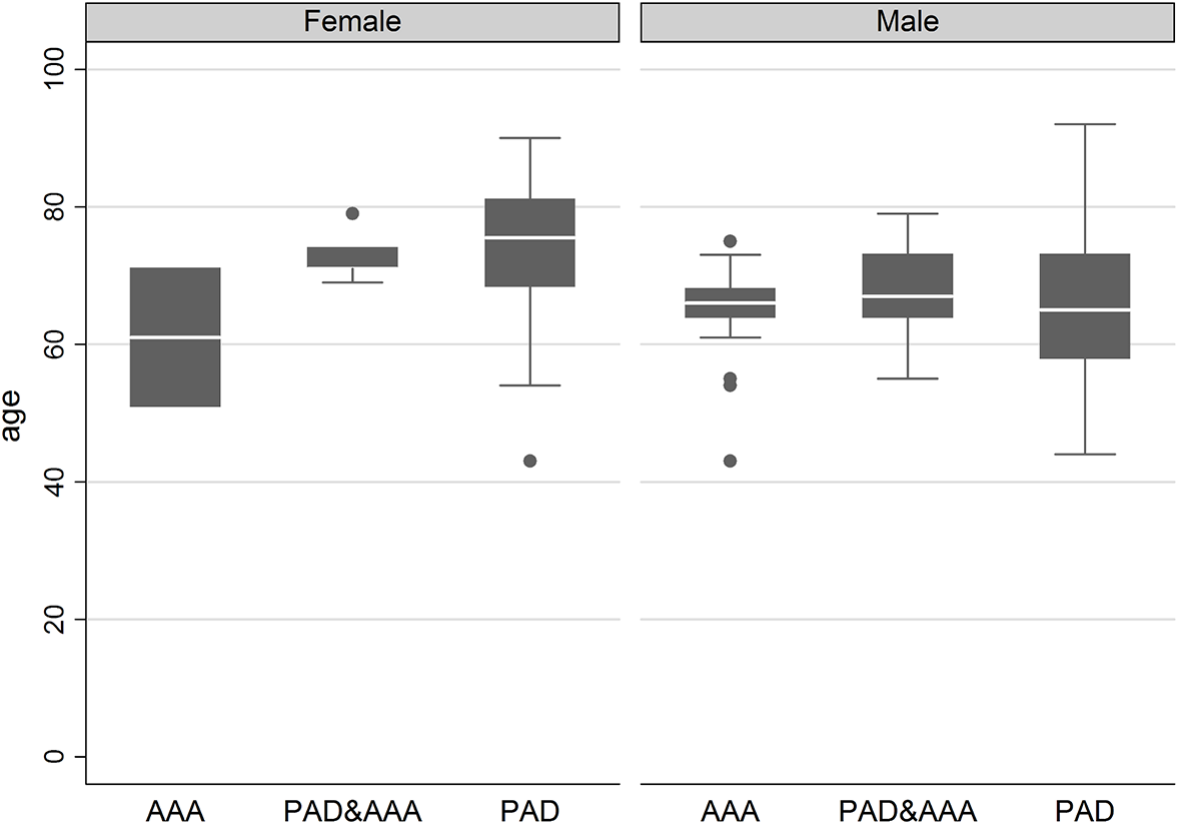
Distribution of Age by Gender and Diagnoses.

Fig 1 illustrates the diagnoses by gender and age. The median age of female patients with aneurysm was 60 years, while the median age of male patients with aneurysm was 68 years. For PAD, the median age of female patients was 75 years, while the median age of male patients was 65 years. Regarding the minimum and maximum ages, there were outliers for female patients with PAD and for male patients with aneurysm.

### Analysis of cAIx according to diagnosis, age and gender

Table 2 presents cAIx values according to patient characteristics. The cAIx was significantly higher in female patients (p = 0.00). The cAIx increased with age, with a significant difference between the three age groups (p = 0.01). It was not correlated with BMI (p = 0.93), but was significantly lower for taller patients (p = 0.00).

**Table 2:** Comparison of cAIx by Patient Characteristics.

		AIx (%)	N	*p*-value
**Gender**	Female	35.24 ± 6.05	55	0.00
	Male	28.44 ± 8.18	129	
**Age (years)**	<65	28.01 ± 8.23	60	0.01
	65–79	31.10 ± 8.03	102	
	>80	34.32 ± 7.19	22	
**BMI**	<25	30.60 ± 8.17	83	0.93
	25–30	30.50 ± 8.09	68	
	>30	30.10 ± 8.77	33	
**Height (cm)**	<160	36.75 ± 6.07	19	0.00
	160–175	30.43 ± 7.54	120	
	>175	28.00 ± 9.36	45	

Notes: The table shows the sample means of the cAIx@75 (heart rate 75 bpm). The p-values refer to the Kruskal-Wallis test without ties, used to determine whether cAIx was significanlty different for gender, age, BMI and height.

Table 3 illustrates cAIx results according to patient groups and diagnosis. There was a difference in cAIx between diagnoses: patients suffering from PAD-only had higher average cAIx values than the other groups, however this was significant at the 10% level only (p = 0.06). We did not see significant differences in cAIx between the three patient groups when split up by age and gender. However, patient groups by age and gender were not “matched” well due to the low number of observations, which may have affected results and also made it impossible to conduct significance tests for all age-gender categories (n/a values).

**Table 3:** Comparison of cAIx by Diagnosis, Gender and Age.

		Aneurysm	PAD	Aneurysm and PAD	Kruskall-Wallis test
		mean cAIx @heart rate 75 (%)	mean cAIx @heart rate 75(%)	mean cAIx @heart rate 75 (%)	*p*-value
**All**		27.28 ± 9.47	31.40 ± 7.80	28.84 ± 8.46	0.06
**Female**	<65	31.71 ± 6.16	34.13 ± 7.60	n/a	1.00
	65–79	40.42 ± 6.16	36.00 ± 4.97	33.34 ± 6.47	0.37
	>80	n/a	35.43 ± 6.63	n/a	n/a
**Male**	<65	23.90 ± 9.70	28.24 ± 8.00	22.92 ± 5.83	0.18
	65–79	27.23 ± 9.48	29.37 ± 7.60	30.02 ± 8.79	0.59
	>80	n/a	32.11 ± 7.58	n/a	n/a

Notes: The table shows the sample means of cAIx@75 (heart rate 75 bpm). The p-values refer to the Kruskal-Wallis test without ties, which was used to determine whether cAIx differed significantly between diagnoses.

### Analysis of cAIx according to diagnosis and comorbidities

Table 4 summarises cAIx in our study population in regard to vascular comorbidities, with patients grouped according to the number of vascular territories affected by atherosclerosis: PAD and/or AAA, coronary artery disease (CAD), and cerebrovascular disease (CVD). The groupings were similar to those of the REACH Registry [21], but AAA was included in the PAD vascular territory. 95 patients (52%) had no known vascular comorbidity, 71 (38%) had either concomitant CAD or CVD, and 18 patients (10%) had all vascular beds affected. Differences in cAIx between these 3 groups were not found to be significant.

**Table 4:** Comparison of cAIx by Comorbidities.

	cAIx@heart rate 75 (%)	Number of patients
(1): PAD and/or AAA	30.67 ± 8.86	95
(2): PAD and/or AAA and CAD or CVD	30.99 ± 7.64	71
(3): PAD and/or AAA and CAD and CVD	27.43 ± 6.28	18

### Multivariate analyses to explore factors influencing cAIx

Our initial regression analysed differences between patients and a control group of healthy individuals, testing whether cAIx in the AAA, PAD and concurrent AAA & PAD patient groups was significantly different from cAIx in an unmatched control group of healthy individuals (Table 5). cAIx in all three patient groups was significantly different from the control group (p < 0.05 in all cases), with age, gender and heart rate taken into account. Age and gender had significant effects on cAIx. We adjusted cAIx for heart rate 75 using the above mentioned transfer function [20], therefore heart rate was not significant and was omitted from subsequent analyses.

**Table 5:** Differences in cAIx between patients and a control group of healthy individuals.

R^2^ = 0.93, N = 202				
Patient group or parameter	Coefficient	SE	Beta	*p*-value
AAA	10.48	4.24	0.30	0.014
PAD	9.58	3.89	0.43	0.015
PAD & AAA	9.27	4.14	0.33	0.026
Age	0.43	0.56	0.56	0.000
Sex	-3.18	1.32	-0.14	0.017
Heart rate	-0.52	0.37	-0.54	0.158

Notes: The table shows results from multivariate linear regression analysis. SE denotes the standard errors of regression coefficients. The dependent variable is cAIx@75 (heart rate 75 bpm)

Table 6 presents results of stepwise multivariate analyses of differences in cAIx between patient groups with differing diagnoses. Model 1 showed that when holding basic characteristics constant (age, gender, height and weight), the “diagnosis”—categorised as 1 (AAA), 2 (AAA & PAD) or 3 (PAD)—significantly influenced cAIx. Model 2 added controls for cardiovascular risk factors to the ‘basic characteristics’, namely for hypertension, smoking habits and diabetes: the significant positive effect of the diagnosis remained robust. Model 3 included the ‘basic characteristics’ and focused on comorbidities. Coronary heart disease had a significant negative effect on cAIx, and the positive effect of diagnosis remained significant, however at the 10% level only (p = 0.063). Cerebrovascular disease was positively correlated with cAIx, but not significantly. Model 4 complemented Model 3, as it was not possible to include comorbidities and medication in the same model. Model 4 showed a significant negative effect of calcium channel blockers on cAIx (a lower and therefore improved cAIx value), as well as a borderline significant negative effect of diuretics on cAIx, corroborating the significant positive effect of diagnosis. In summary, Models 1 to 4 demonstrated that patients with PAD only, or AAA plus PAD, had a significantly higher cAIx than patients with AAA, even after controlling for a rich set of confounding factors.

**Table 6:** Differences in cAIx between patient groups.

Parameters	Coefficient	SE	Beta	p-value
**Model 1:** R^2^ = 0.99, N = 181				
Diagnosis	0.09	0.34	0.21	0.006
Age	0.11	0.00	0.35	0.000
Male	-0.18	0.06	-0.27	0.004
Height	-1.43	0.64	-0.23	0.026
**Model 2:** R^2^ = 0.99, N = 181				
Diagnosis	0.08	0.04	0.18	0.029
Hypertension	-0.07	0.07	-0.09	0.256
Diabetes	-0.11	0.06	-0.16	0.042
Smoker	0.08	0.04	0.12	0.140
*further controls*: *age*, *sex*, *height*, *weight*.				
**Model 3:** R^2^ = 0.99, N = 181				
Diagnosis	0.07	0.04	0.15	0.063
Coronary Heart Disease	-0.12	0.05	-0.18	0.023
Cerebrovascular Disease	0.06	0.06	0.08	0.323
*further controls*: *age*, *sex*, *height*, *weight*.				
**Model 4:** R^2^ = 0.99, N = 181				
Diagnosis	0.08	0.03	0.17	0.023
Statins	0.02	0.06	0.02	0.753
ACE / AT II	0.02	0.05	0.04	0.666
β-Blockers	-0.07	0.05	-0.12	0.139
CA-Ant.	-0.11	0.05	-0.17	0.032
Diuretics	-0.09	0.05	-0.14	0.103
*further controls*: *age*, *sex*, *height*, *weight*.				
**Model 5:** R^2^ = 0.99, N = 181				
Diagnosis	0.09	0.03	0.19	0.014
Age	0.01	0.00	0.39	0.000
Male	-0.12	0.06	-0.17	0.064
Height	-1.95	0.62	-0.31	0.002
Diabetes	-0.10	0.05	-0.14	0.064
Coronary Heart Disease	-0.10	0.05	-0.16	0.043
CA-Ant.	-0.14	0.05	-0.21	0.004
**Model 6:** R^2^ = 0.99, N = 181				
PAD	0.16	0.07	0.24	0.034
PAD & AAA	0.10	0.08	0.12	0.248
Age	0.01	0.00	0.39	0.000
Male	-0.12	0.06	-0.18	0.056
Height	-1.95	0.63	-0.31	0.002
Diabetes	-0.10	0.05	-0.14	0.074
Coronary Heart Disease	-0.11	0.05	-0.17	0.034
CA-Ant.	-0.14	0.05	-0.21	0.005

Notes: The table shows results from multivariate linear regression analyses. SE denotes the standard errors of regression coefficients. The dependent variable is cAIx@75 (heart rate 75 bpm)

Models 5 and 6 tested the robustness of these results. Model 5 included all variables which were significant in Models 1 to 4, and diagnosis remained significant. Model 6 used an alternative specification of diagnosis: rather than including one ordinal variable which ranged from 1 to 3, it included two binary variables. The first took the value one if the patient suffered from PAD only and was zero otherwise; the second variable took the value one if the patient suffered from PAD and AAA. Results were therefore expressed relative to patients with AAA only. The model showed that cAIx was significantly higher for patients suffering from PAD only (p-value: 0.034) compared to patients suffering from AAA only.

In order to test robustness further, a z-score Index was calculated (see S1 Text).

## Discussion

To the best of our knowledge, this is the first study to compare cAIx in patients with PAD and AAA. We found that the presence of peripheral arterial disease and / or abdominal aortic aneurysm was associated with an elevated cAIx and was highest in patients with PAD only, followed by patients with PAD and concomitant AAA, and lowest in patients with AAA without evidence for PAD. Although this study was based on a fairly low number of observations (n = 184), and the patient groups differed in regard to number, age and sex, the results were consistent across a comprehensive range of analyses that took account of confounding factors.

Previous studies have focused either on patients with PAD or AAA only. Several groups have reported an association between cAIx and PAD. Khalegi and Kullo found that cAIx was higher in patients with asymptomatic PAD compared to age- and sex-matched controls [22]. Brewer et al. [23] observed that lower cAIx was associated with more physical exercise (longer walking distances) in patients with PAD. In a previous study, we similarly found a significant correlation between ankle-brachial index and aortic augmentation index in PAD patients, and significant correlation for these patients between ABI and subendocardial viability ratio (another non-invasive hemodynamic marker derived from pulse wave analysis) [24]. In addition, we found that lower limb revascularization was associated with a 10% lowering in cAIx after 3 months when compared to a PAD control group treated conservatively [25]. Taken together, the existing literature lends support to the conclusion of our current study, that cAIx is a marker for arterial stiffness and is abnormally elevated in PAD.

Concerning AAA, previous research does not present a similarly clear-cut picture. Lee et al. [26] studied a sample of 51 patients and found a significantly lower PWV and higher cAIx in patients with AAA compared to controls, arguing that these were not reliable markers in patients with AAA. However, using a study group of only 19 patients, Moloney et al. found that cAIx improved after both open and endovascular repair implying a better compliance and further indicating that arterial stiffness markers were positively influenced by surgery [27]. In light of evidence that the rupture rate for AAA increases with aneurysm growth rate, Ruegg et al. did not find that cAIx differed significantly between fast and slow progressors [28].

The cAIx has been shown to be influenced by many different factors, such as: heart rate [29], age [30], gender (regardless of height) [31–34], height [35], ethnicity [36], risk factors and comorbidities (hypercholesterolemia [37], diabetes [38], renal failure [39]), and even living habits such as time spent watching television [40]. Our data conformed with results from previous studies in many respects, for example, cAIx in our patient group was strongly dependent on age (the difference between the three age groups in our patient sample was significant), gender (higher among females) and subject height. Also in line with previous research, cAIx was not associated with BMI [35]. Wilkinson et al. [29] demonstrated a negative effect of heart rate on cAIx. In our study, heart rate also had a significant effect, but our preferred specification was cAIx@75 (heart rate 75 bpm) (Table 5).

As expected, we found that cAIx was elevated in patients with PAD compared to unmatched controls, consistent with Catalano et al.’s study of PWV in PAD patients [41]. cAIx was also elevated in patients with AAA compared to our unmatched controls. This demonstrates that cAIx can be an indicator of both occlusive and aneurysmatic arterial disease.

Our novel contribution to the literature is in analyzing the relative elevation of cAIx in patient groups with different diagnoses. We showed that cAIx depended on the pattern, i.e. obstructive versus aneurysmal, of the disease. Given the confirmed sensitivity of cAIx with regard to patient characteristics, our preferred method of analysis was matching by age and gender. However we had very few observations in some of the groups, given our sample of 184 patients and that, for example, PAD is more common in older patients and AAA is rarer in females. Nevertheless, we found cAIx to be higher in patients with PAD than in those with AAA, especially in the well-matched male group (this relative elevation was corroborated using a regression analysis that controlled for further patient characteristics). Taken together the results were consistent across a range of analyses, indicating that the findings were robust.

There were, however, some recognised weaknesses in the statistical analyses employed. Most importantly, the low number of observations and the significant overlap between patient groups led to multi-collinearity and therefore did not allow us to control for all patient characteristics simultaneously. Furthermore, due to the low number of observations and shared comorbidities, risk factors and medication, for both PAD and AAA, we cannot exclude the possibility of coincidental significance for certain patient characteristics.

Our results, and those from other studies on cAIx in PAD and AAA patients, need to be considered in the context of vascular ageing and disease-related changes in vascular wall properties, and should be evaluated for clinical relevance. Age- and disease-related stiffening of the elastic arterial wall increases PWV and thereby impacts the antegrade and retrograde pulse waves similarly, resulting in central pressure augmentation. Based on the physiology of pulse wave propagation and reflexion, both an obstructive as well as an aneurysmatic vascular disease will have an impact on central aortic pressure hemodynamics. The central pressure augmentation has an unfavourable influence on systolic cardiac afterload and diastolic myocardial perfusion, both of which may be a factor for cardiovascular events such as stroke and myocardial infarction. Prognosis in both PAD and AAA patients is determined by cerebrovascular and cardiac events, as well as by disease-specific severe vascular complications such as gangrene and sepsis, or aneurysm rupture. Blood pressure management is an important preventative measure for all of these vascular complications.

Central arterial blood pressure does not necessarily correspond to brachial arterial pressure due to the pressure pulse amplification phenomenon in the vascular bed. It has been shown that central pressures and arterial stiffness indices such as the central augmentation index (cAIx) might be of greater relevance than peripheral pressures in the pathogenesis of cardiovascular events [42,43, 15, 44]. Hence, peripheral blood pressures might also not fully reflect the effects of medical treatment on blood pressure and cardiovascular risk markers, as shown in the sub-study of the ASCOT (CAFE) trial [45–46]. A better understanding of central hemodynamic markers and their predictive value for all-cause-mortality and cardiovascular risk, as well as their utility as follow-up markers for optimal blood pressure management, could lead to a new understanding of cardiovascular risk assessment. This applies in particular to patients with a known high-risk profile such as PAD and AAA.

In conclusion, our study found that central aortic pressure augmentation was elevated in patients with PAD and AAA. cAIx was higher in PAD patients than in AAA patients. The elevation itself was not surprising given that atherosclerosis causes increased arterial stiffness. The relative degree of elevation in cAIx was significant but only descriptive. Therefore, in practice, when using cAIx for risk stratification, the sensitivity of cAIx with regard to vascular disease patterns has to be considered.

## Supporting Information

S1 Data (DTA)

S1 Table Characteristics of unmatched control group. (PDF)

S1 Text z-score. (PDF)
