# Supplementary material for: Risk Stratification of Patients with Peripheral Arterial Disease and Abdominal Aortic Aneurysm Using Aortic Augmentation Index
Source: PLoS One. 2015 Oct 9;10(10):e0139887. doi: 10.1371/journal.pone.0139887 (PMC4599890; doi:10.1371/journal.pone.0139887)
Supplement: S1 Table — (PDF) [file pone.0139887.s002.pdf]

S2 Table: Characteristics of unmatched control group

Age, years	35.00 ±10.74
Male, n (%)	6 (33)
Cardiovascular comorbidities	None
Cardiovascular risk factors	None
Medication	None
cAIx@ heart rate 75 (%)	11.57±12.75

Notes: n denotes the number of observations
