# Supplementary material for: Risk Stratification of Patients with Peripheral Arterial Disease and Abdominal Aortic Aneurysm Using Aortic Augmentation Index
Source: PLoS One. 2015 Oct 9;10(10):e0139887. doi: 10.1371/journal.pone.0139887 (PMC4599890; doi:10.1371/journal.pone.0139887)

S3. z –score

As a further step to test the robustness of our results, we standardized the cAIx by calculating a *z*-score. The *z*-score was calculated as cAIx minus the mean of the population divided by the standard deviation, and it took account of heterogeneities between patients such as age and gender. A *z*-score of zero would have implied that cAIx did not differ from the population mean. Figure 2 (below) presents the results as a box plot: there was a negative *z*-score for patients with AAA, and a slightly negative *z*-score for patients with both PAD and AAA. These results indicated that the cAIx was lower for patients with aneurysm than for patients with PAD.

Figure 2

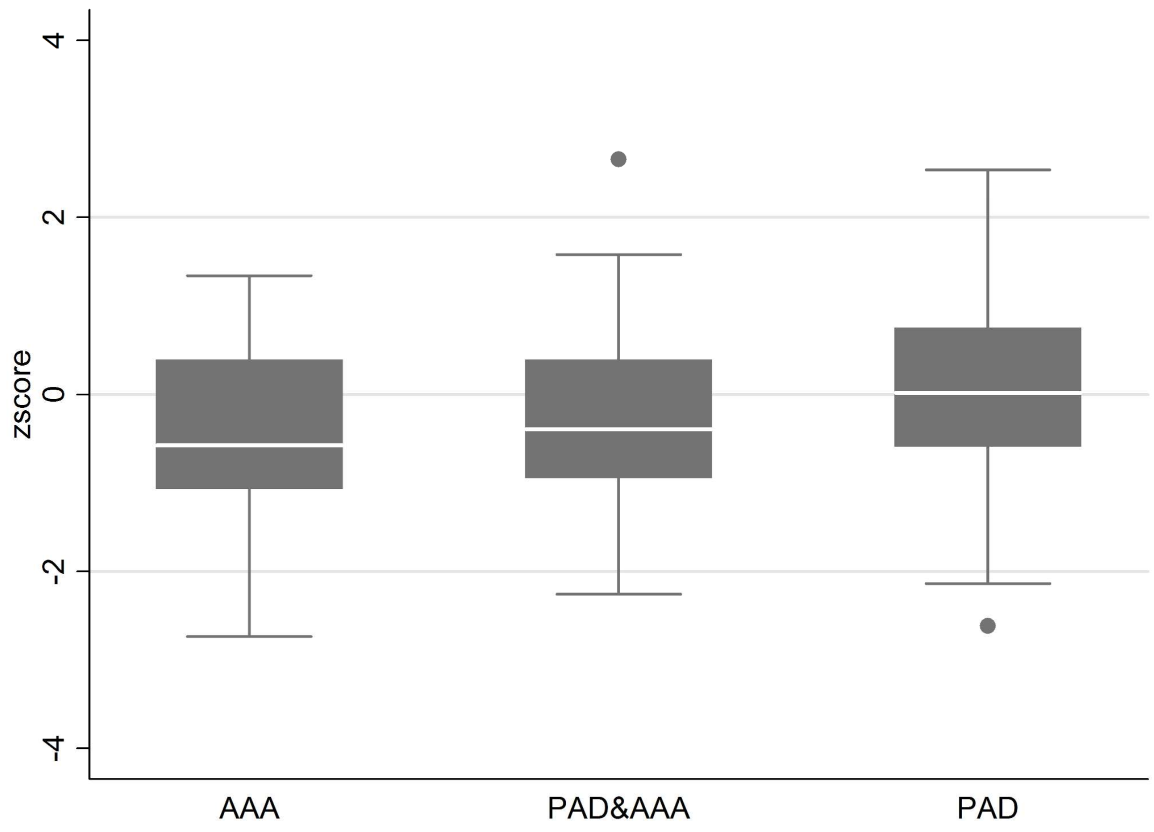

Supplement: S1 Text — (PDF) [file pone.0139887.s003.pdf]
